# Incarcerated femoral hernia containing the ipsilateral fallopian tube without ovarian involvement: A rare case report

**DOI:** 10.1016/j.ijscr.2025.111079

**Published:** 2025-02-24

**Authors:** Hiba Ben Hassine, Ferdaoues Ouertani, Faiez Boughanmi, Midani Touati, Ibtissem Korbi, Faouzi Noomen

**Affiliations:** Department of Visceral Surgery, Fattouma Bourguiba Hospital, Monastir, Tunisia

**Keywords:** Incarcerated femoral hernia, Fallopian tube, Hernia repair, Emergency surgery

## Abstract

**Introduction:**

Femoral hernias, though less common than inguinal hernias, have a higher propensity for incarceration and strangulation, often leading to significant morbidity and mortality. They are more prevalent in women, and their clinical diagnosis remains challenging.

**Presentation of case:**

This case report describes a 35-year-old woman presenting with an incarcerated right femoral hernia containing the ipsilateral fallopian tube alone, without the ovary. Emergency surgery revealed the fallopian tube within the hernia sac without ischemic changes, and repair was successfully performed using the McVay operation.

**Discussion:**

Femoral hernias are rare but prone to complications like incarceration and strangulation. Isolated fallopian tube incarceration without ovarian involvement is extremely uncommon and challenging to diagnose, often requiring imaging for confirmation. Early surgical intervention, such as the McVay operation, is essential to prevent morbidity and ensure favorable outcomes.

**Conclusion:**

This case highlights the importance of early diagnosis and appropriate surgical intervention in managing rare presentations of femoral hernias to prevent complications.

## Highlights

• Femoral hernias account for 2–8 % of hernias, with high risk of incarceration.
• Isolated fallopian tube in femoral hernia sac is extremely rare and poorly documented.
• Preoperative diagnosis is challenging; imaging like Doppler ultrasound is essential.
• The McVay operation is a reliable technique for femoral hernia repair without mesh.
• Early surgical intervention is crucial to avoid strangulation and complications.

## 1 Introduction

Femoral hernias, though less common than inguinal hernias, are frequently associated with incarceration, leading to notable morbidity and mortality. They are more prevalent in women and exhibit a higher tendency for incarceration compared to other abdominal hernias. This predisposition is attributed to the narrow defect of the femoral ring and its rigid ligamentous boundaries. Incarceration of a femoral hernia containing the fallopian tube, particularly without the ovary, is an exceptionally rare occurrence [1]. This case study aims to report a unique presentation of a right incarcerated femoral hernia containing the ipsilateral fallopian tube alone within the hernia sac, without the involvement of the ovary. This report adheres to the SCARE guidelines 2023 [2].

## 2 Patient and observation

A 35-year-old woman presented to the emergency department complaining of a three-day history of right groin swelling which gradually became tender to palpation during the last 48 h. Twenty-four hours before her admission to our department, the patient was examined by her gynecologist, who did not find any obvious gynecological disorder.

Upon presentation to our department, her vital signs were stable: temperature 37 °C, pulse rate 76 beats/min, blood pressure 110/85 mmHg, and respiratory rate 16/min. Physical examination revealed swelling right inguinal region measuring approximately 4 cm × 3 cm in size, tense, tender without impulse on cough, rebound tenderness was absent and the rest of the abdomen was soft without distension. The patient's BMI was 22.4 kg/m^2^. She had no known occupational risk factors, such as heavy lifting. The patient had no significant past medical or surgical history. She was not on any regular medications, and there was no family history of hernias or connective tissue disorders. Laboratory findings included leukocytosis (12,700/mm^3^) and an elevated C-reactive protein level (6 mg/dL).

Based on these findings, the diagnosis of an incarcerated femoral hernia was made and the patient underwent emergent operation.

**Fig. 1:**
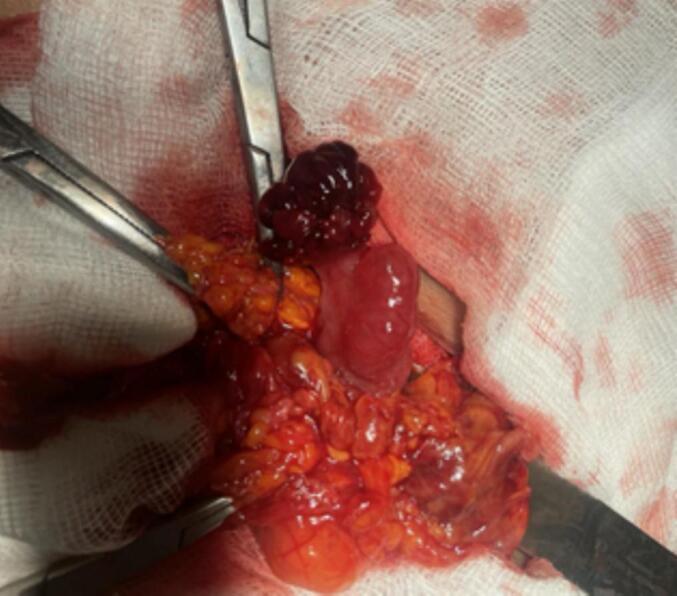
The open femoral hernia sac with its uncommon content the ipsilateral fallopian tube.

**Fig. 2:**
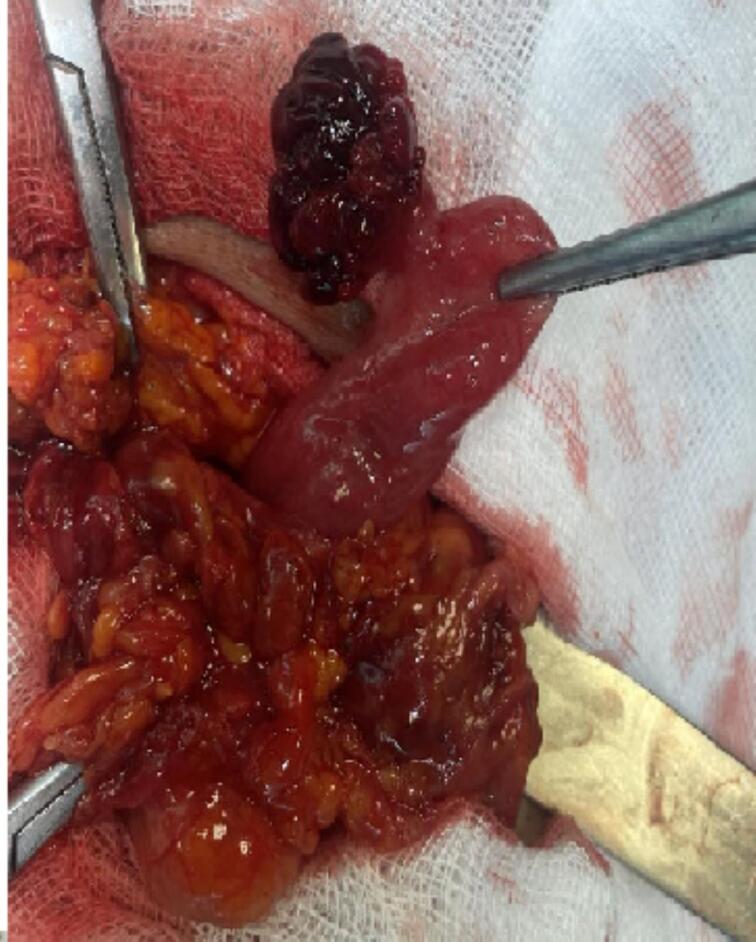
The uterine tube with its mesosalpinx, while the right ovary lies within the abdominal cavity.

Intraoperatively, the hernia was found passing posterior to the inguinal ligament, medial to the femoral vein, and lateral to pubic tubercle to saphenous opening then anteriorly and then cephalad over the inguinal ligament. The sac was opened to find fluid and a fallopian tube with infundibulum and fimbriae. After aspirating the fluid, a strangulated femoral hernia sac containing the right fallopian tube was detected (Fig. 1, Fig. 2). No signs of ischemic damage were detected and The fallopian tube was carefully reduced into the abdominal cavity. The hernia sac was closed at its base, with the redundant portion excised. McVay's operation was performed without the use of a polypropylene plug.

The patient's postoperative course was uneventful, and she was discharged on the second postoperative day. The patient was followed up at three and six months postoperatively, with no signs of recurrence or complications. This has been added to the discussion.

## 3 Discussion

Femoral hernias account for approximately 2 % to 8 % of all hernias in adults, accounting for fewer than 5 % of all groin hernias. They are significantly more common in women, with an incidence ratio of 10:1 compared to men. They are associated with higher rates of acute complications. Approximately 36 % of femoral hernias present with incarceration and many are not diagnosed until the patient presents with incarceration, strangulation, or bowel obstruction because of their narrow neck and rigid ligamentous borders, often necessitating emergency surgical intervention [1].

Incarcerated femoral hernia containing the fallopian tube is an exceptionally rare condition, primarily due to the normal anatomical position of the fallopian tube below the femoral triangle and the absence of any embryological connection between these structures [3]. The first documented case of an inguinal hernia containing the uterine tube alone, without the ovary, was reported by Voigt in 1809 [4]. Conversely, the presence of the ovary within the sac of a femoral hernia is relatively more common.

The preoperative diagnosis of femoral hernia remains a significant challenge, with reported diagnostic accuracy ranging from 25 % to 40 %. [5]. The differential diagnosis includes indirect inguinal hernia, enlarged lymph nodes, hydrocele of the canal of Nuck, cord lipoma, obturator hernia, and psoas abscess [6]. Difficulties in diagnosis are often attributed to the rarity of the condition, limited experience of the surgeon, and insufficient physical examination.

Doppler ultrasonography can be a valuable tool for assessing the blood flow of the hernia's contents and identifying signs of strangulation. In cases where bowel loops are present within the hernia sac, findings such as an edematous bowel wall or absent peristalsis may indicate strangulation [5].

Various surgical techniques are available for femoral hernia repair, including the McVay operation, the polypropylene plug mesh technique, and laparoscopic approaches. The laparoscopic method offers the advantage of allowing thorough inspection of incarcerated or irreducible contents, identifying ischemic changes, and minimizing the risk of missing any coexisting pathology. In the presented case, the McVay operation was utilized for repair.

The recurrence rate following femoral hernia repair varies depending on factors such as the surgical technique used and whether the surgery was elective or emergency. Studies have reported recurrence rates ranging from approximately 1 % to 10 %. A systematic review found an overall recurrence rate of about 4 % after open primary femoral hernia repairs, with higher rates observed in non-mesh techniques and emergency surgeries.

Additionally, recurrence rates can be influenced by factors such as the hernia site, type of repair, and clinical circumstances. [7]. Notably, the introduction of the polypropylene plug mesh technique has significantly reduced recurrence rates, offering improved long-term outcomes [1].

## 4 Conclusion

A femoral hernia may present as a swelling in the inguinal region and should always be kept in mind whenever a female patient presents with such a swelling. As strangulation is the most common complication of femoral hernias they should be suspected, diagnosed, and managed on an emergency basis to prevent gangrene of herniating contents.

## Consent

Written informed consent was obtained from the patient for publication of this case report and accompanying images. A copy of the written consent is available for review by the Editor-in-Chief of this journal on request.

## Ethical approval

The study was approved by Ethics Committee of Hospital Fattouma Bourguiba Monastir.

## Ethical approval

Ethical approval is exempt/waived at our institution.

Ethics approval is not required for case reports deemed not to constitute research at our institution.

## Guarantor

Hiba Ben Hassine.

## Author contributions

All the authors participated in the treatment of the patients, writing, and approving the manuscript.

## Funding

This research received no specific grant from the public, commercial, or not-for-profit sectors.

## Declaration of competing interest

No conflict of interest to disclose. The authors declare no competing interest. The study did not receive any sources of support or funding.
